# Effect of *SIRT1* gene single-nucleotide polymorphisms on susceptibility to type 1 diabetes in a Han Chinese population

**DOI:** 10.1007/s40618-023-02190-5

**Published:** 2023-09-11

**Authors:** J. Li, Y. Yang, Y. Xia, S. Luo, J. Lin, Y. Xiao, X. Li, G. Huang, L. Yang, Z. Xie, Z. Zhou

**Affiliations:** 1 National Clinical Research Center for Metabolic Diseases, Key Laboratory of Diabetes, Immunology (Central South University), Ministry of Education, and Department of Metabolism and Endocrinology, The Second Xiangya Hospital of Central South University, Changsha, 410011 Hunan China; 2 Department of Laboratory Medicine, The Second Xiangya Hospital of Central South University, Changsha, China

**Keywords:** *SIRT1* gene, Single-nucleotide polymorphisms, Type 1 diabetes, Protein tyrosine phosphatase autoantibody, Han Chinese population

## Abstract

**Aims:**

SIRT1 deficiency has been associated with diabetes, and a variant of the *SIRT1* gene has been found to be involved in human autoimmune diabetes; however, it is unclear whether this genetic variation exists in Han Chinese with type 1 diabetes (T1D) and whether it contributes to development of T1D. Therefore, we aimed to explore the association of the *SIRT1* gene single-nucleotide polymorphisms (SNPs) rs10997866 and rs3818292 in a Han Chinese population with T1D.

**Methods:**

This study recruited 2653 unrelated Han Chinese individuals, of whom 1289 had T1D and 1364 were healthy controls. Allelic and genotypic distributions of *SIRT1* polymorphisms (rs10997866 and rs3818292) were determined by MassARRAY. Basic characteristics, genotype and allele frequencies of selected SNPs were compared between the T1D patients and healthy controls. Further genotype–phenotype association analysis of the SNPs was performed on the T1D patients divided into three groups according to genotype. Statistical analyses included the chi-square test, Mann‒Whitney U test, Kruskal‒Wallis H test and logistic regression.

**Results:**

The allelic (G vs. A) and genotypic (GA vs. AA) distributions of *SIRT1* rs10997866 were significantly different in T1D patients and healthy controls (*P* = 0.039, *P* = 0.027), and rs10997866 was associated with T1D susceptibility under dominant, overdominant and additive models (*P* = 0.026, *P* = 0.030 and *P* = 0.027, respectively). Moreover, genotype–phenotype association analysis showed the GG genotype of rs10997866 and the GG genotype of rs3818292 to be associated with higher titers of IA-2A (*P* = 0.013 and *P* = 0.038, respectively).

**Conclusion:**

*SIRT1* rs10997866 is significantly associated with T1D susceptibility, with the minor allele G conferring a higher risk of T1D. Moreover, *SIRT1* gene rs10997866 and rs3818292 correlate with the titer of IA-2A in Han Chinese individuals with T1D.

## Introduction

Type 1 diabetes (T1D) is an organ-specific chronic autoimmune disease characterized by insulin deficiency and hyperglycemia. Although the mechanisms of T1D development and progression are not completely clear, it is currently believed to be a complex genetic disease resulting from interaction of a series of genetic and environmental factors [1, 2]. Although most (approximately 70%) T1D patients do not have a positive family history, the family genetic tendency of T1D, a polygenic disease, is obvious [3]. In addition, the comorbidity risk of T1D between siblings is higher than that between parents and offspring among first-degree relatives, indicating that T1D has strong genetic susceptibility [4].

Previous studies have shown that the human leukocyte antigen (HLA) region contains the main susceptibility genes for human T1D, with *HLA II* genes contributing approximately 50% of genetic risk [5]. Genome-wide association studies (GWASs) have identified over 60 non-*HLA* genes related to risk of T1D, and these genes contribute to inheritance of the disease through small genetic effects resulting from their various combinations [5]. In general, understanding the association of non-*HLA* genes with T1D is helpful for identifying potential targets for treatment and for developing specific immune-related interventions.

SIRT1 is a kind of NAD^+^-dependent histone deacetylase that plays an important role in energy metabolism, apoptosis and aging [6]. A variety of studies have shown that SIRT1 regulates inflammation, gluconeogenesis, lipolysis, β cell survival and glucose-dependent insulin secretion by interacting with many histone and nonhistone substrates, directly or indirectly affecting the occurrence and development of diabetes, especially type 2 diabetes (T2D) [7, 8]. A series of studies have highlighted the influence of single-nucleotide polymorphisms (SNPs) of the *SIRT1* gene on various aspects of T2D, including its risk, insulin resistance, diabetes-related traits and associated complications [9], for example, T2DM-related coronary heart disease (rs16924934 and rs3818291 in the Han Chinese population [10] and rs7896005 [10, 11]) and diabetic kidney disease (rs10823108, rs3818292 and rs7069102) [12–14]. Furthermore, rs7895833 and rs1467568 of *SIRT1* are involved in fetal programming during malnutrition, thus affecting T2D risk later in life [15].

Importantly, *SIRT1* plays a role in the disease susceptibility of T2D and its associated complications, and some studies have suggested that *SIRT1* gene variants influence autoimmune diseases. *SIRT1* promoter rs3758391 was shown to modify the morbidity risk due to systemic lupus erythematosus (SLE), and the T allele acts as a risk factor for progression of nephritis and a higher SLE disease activity index [16]. Recently, researchers have found that a novel rare variant of the *SIRT1* gene (Leu107Pro) is responsible for the autoimmune diabetes phenotype, providing a novel idea and direction for further exploration of *SIRT1* single-gene variants related to the pathogenesis of T1D [17].

The present study extends the only report thus far in T1D by investigating the association between *SIRT1* gene polymorphisms and the clinical characteristics of T1D patients in a cross-sectional analysis. The basic characteristics and allele and genotype frequencies of selected SNPs in T1D patients and healthy controls in the Han Chinese population were analyzed to confirm whether there is a correlation between *SIRT1* variants and risk of T1D.

## Methods

### Patients and healthy subjects

The participants in this study consisted of 1289 T1D patients from the endocrine inpatient and outpatient clinic of the Second Xiangya Hospital of Central South University (Changsha, China) and 1364 healthy controls recruited from the same hospital physical examination center and epidemiological investigations. All participants were from the Han Chinese population and provided signed informed consent, and the ethics committee of the Second Xiangya Hospital of Central South University approved this study.

A standard diagnosis of T1D was first made in accordance with the 1999 WHO diagnostic criteria for diabetes and with acute onset, and insulin injections were needed within half a year of diagnosis to achieve adequate glucose control. In addition, we tested for islet self-antibodies, including glutamic acid decarboxylase autoantibody (GADA), protein tyrosine phosphatase autoantibody (IA-2A) and zinc transporter eight antibody (ZnT8A). Patients with serum positivity for at least one were included in the study. We excluded patients with T2D, gestational diabetes, specific types of diabetes and other autoimmune diseases.

The healthy controls with a fasting plasma glucose (FPG) level < 5.6 mmol/L and a 2-h postprandial plasma glucose (PPG) level < 7.8 mmol/L according to the 75 g oral glucose tolerance test (OGTT) were recruited through normal clinical examination results. To reduce bias, we strictly followed the admission rules of the control group, which not only included the nondiabetes Han Chinese population but also excluded people with chronic diseases and endocrine diseases, other types of autoimmune diseases, family history of diabetes, and malignant tumors.

### Information collection

Information for all subjects, including age, sex, diagnosis and treatment process, current medical history, past and family history, height and weight, was measured and collected by trained physicians. Body mass index (BMI) was calculated as weight/height^2^ (kg/m^2^). Levels of FBG, PPG and other biochemical indicators were determined via automated liquid chromatography and chemiluminescence methods; levels of GADA, IA-2A and ZnT8A were detected via radioligand binding assays. Fasting C-peptide (FCP) and 2-h postprandial C-peptide (2hCP) levels were assessed among individuals diagnosed with T1D after ameliorating glucotoxicity. The cutoff point for deficient beta-cell function was defined as FCP or 2hCP < 16.5 pmol/L. Impaired beta-cell function was indicated as FCP or 2hCP between 16.5 and 200 pmol/L, and patients with both FCP and 2hCP exceeding 200 pmol/L were defined as having preserved beta-cell function, as previously reported. All biochemical indicators were determined in the Department of Metabolism and Endocrinology, The Second Xiangya Hospital of Central South University.

### DNA preparation and genotyping assay

Approximately 4 mL of peripheral blood from all participants was collected through venipuncture and placed in tubes containing sodium ethylenediaminetetraacetic acid. Genomic DNA was extracted from the blood samples using a GeneNode Genomic DNA Extraction kit (Genenode Biotech Co., Ltd., Beijing), and the quality of the DNA was assessed using agarose gel electrophoresis (Bio-Rad Company, USA). The DNA samples were stored at -80 °C before being analyzed for SNP genotype using MassARRAY technology (Agena, MassARRAY® Analyzer 4) at BGI Genomics (Beijing Genomics Institute, Shenzhen, China).

### Single-nucleotide polymorphism selection

**Fig. 1:**
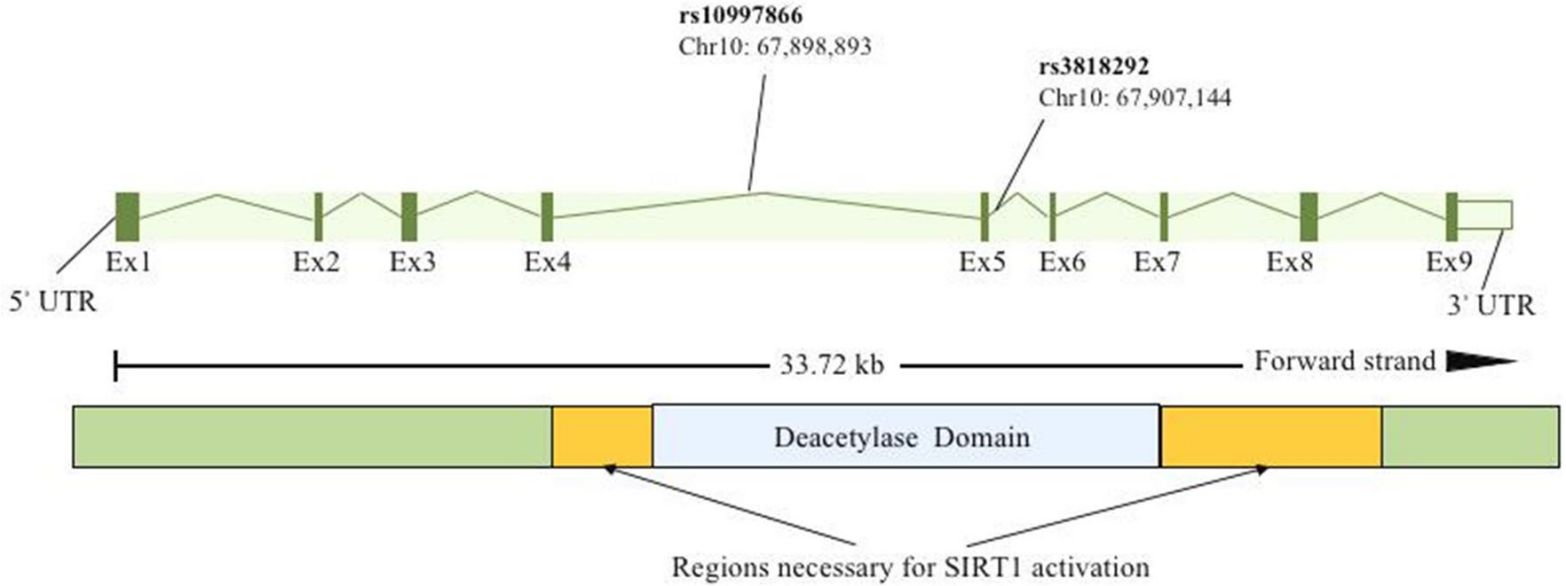
Detailed schematic diagram of two *SIRT1* gene polymorphisms (rs10997866 and rs3818292)

We chose two tagSNPs, rs10997866 and rs3818292, which both satisfy minor allele frequency (MAF) > 0.05 in the Asian population and are not located in the same linkage region (Fig. 1). The r^2^ between rs10997866 and rs3818292 is 0.084 in the East Asian population (https://ldlink.nci.nih.gov/?tab=home). In addition, these two SNPs of the *SIRT1* gene were selected mainly depending on the loci related to some autoimmune diseases reported in recent years [13, 18].

### Statistical analysis

All statistical analyses were performed using SPSS 26.0 software (IBM Corporation, Armonk, NY, USA). Measurement data conforming to a normal distribution are expressed as the mean ± standard deviation (SD). Continuous variables not normally distributed are presented as the median and interquartile range (IQR), and categorical data are presented as frequencies (percentages). Hardy–Weinberg equilibrium for each SNP in the control population was assessed for further analysis. Student's t test or the Mann–Whitney U test was used to compare differences between groups, and the chi-square test or Fisher's exact test was used to identify differences in proportions between groups. Odds ratios (ORs) and 95% confidence intervals (CIs) were obtained using logistic regression analysis to assess differences in alleles/genotypes between the T1D patients and healthy controls. The Kruskal‒Wallis H test and chi-square test were used to compare the characteristics of different genotypes in T1D patients. A two-tailed *P* < 0.05 was considered statistically significant.

## Results

### Basic characteristics

The basic characteristics of the T1D patients and healthy controls are shown in Table 1. The results demonstrated that the sex ratio did not differ significantly between the two groups (*P* = 0.932). However, significant differences were observed in other basic characteristics, including age, BMI, FBG, and PBG, (all, *P* < 0.05), but not for sex. The healthy controls had higher age and BMI values than the T1D patients (*P* < 0.001 and *P* < 0.001), and the T1D patients had higher levels of FBG and PBG (*P* < 0.001 and *P* < 0.001). Our data also indicated that the enrolled T1D patients had FCP and PCP levels of 88.01 (33.42, 170.00) pmol/L and 173.00 (63.73, 351.60) pmol/L, respectively, and HbA1c levels of 9.7 (7.3–12.7)%.

**Table 1:** Clinical characteristics of T1D patients and healthy controls

Clinical characteristics	T1D patients	Healthy controls	*P* value
N (male/female)	1289 (692/597)	1364 (730/634)	0.932
Age (years)	24 (13, 36)	41 (28, 50)	< 0.001
Age of onset (years)	21.99 (12.00, 34.00)	–	–
BMI (kg/m^2^)	20.03 ± 3.92	22.67 ± 4.32	< 0.001
Duration of T1D (moths)	3.00 (0.40, 14.00)	–	–
FBG (mmol/L)	8.60 (6.01, 13.29)	5.00 (4.60, 5.37)	< 0.001
PBG (mmol/L)	15.38 (10.48, 20.64)	5.70 (4.80, 6.50)	< 0.001
FCP (pmol/L)	88.01 (33.42, 170.00)	–	–
PCP (pmol/L)	173.00 (63.73, 351.60)	–	–
HbA1c (%)	10.20 (7.80, 12.90)	–	–

All data except BMI are presented as the median (interquartile range). The BMI data were consistent with the distribution and are described as the mean ± standard deviation

*N* number of subjects, *BMI* body mass index, *FBG* fasting blood glucose, *PBG* postprandial blood glucose, *FCP* fasting C-peptide, *PCP* postprandial C-peptide, *HbA1c* hemoglobin A1c

### Associations between alleles and genotypes of single-nucleotide polymorphisms in the SIRT1 gene and T1D

All the genotype frequency distributions of the two SNPs (rs10997866 and rs3818292) in our healthy controls were consistent with Hardy–Weinberg equilibrium (HWE) proportions (*P* = 0.335 and *P* = 0.656, respectively), which indicated that the recruited subjects are representative of the population.

The results indicated that *SIRT1* rs10997866 is linked to an increased risk of developing T1D, as the G allele of rs10997866 conferred a higher risk of T1D (OR = 1.18, 95% CI = 1.01–1.37, *P* = 0.039). Moreover, we compared three different genotypes of this SNP locus in pairs. The AA genotype was taken as the reference, with an OR value of 1. The OR value and 95% CI of homozygous and heterozygous minor alleles were calculated. The heterozygous GA genotype of rs10997866 showed a significant association with T1D risk (OR = 1.22, 95% CI = 1.02–1.46, *P* = 0.027), whereas no significant association was found between the homozygous AA genotype and T1D risk (*P* > 0.05). There was no significant difference in the distribution of alleles and genotypes of rs3818292 between the T1D patients and healthy controls (Table 2). Furthermore, the association between the two SNPs of *SIRT1* and T1D susceptibility was examined under various genetic models, including dominant, recessive, overdominant, and additive. The results suggested that only *SIRT1* rs10997866 has a statistically significant association with T1D susceptibility under dominant, overdominant, and additive genetic models (*P* = 0.026, *P* = 0.030 and *P* = 0.027, respectively). Conversely, no statistically significant association was found between *SIRT1* rs3818292 and T1D susceptibility under the four genetic inheritance models (Table 3).

**Table 2:** Frequencies of allele and genotype distributions of rs10997866 and rs3818292 between T1D and healthy controls

SNP	N (%)		*P*	OR (95% CI)
	T1D patients	Controls		
*rs10997866*				
*Allele*				
G	420 (16.4)	375 (14.3)	**0.039**	1.17(1.01–1.36)
A	2146 (83.6)	2247 (85.7)	N/A	1
*Genotype*				
GG	33 (2.6)	31 (2.4)	0.585	1.15(0.70–1.89)
AG	354 (27.6)	313 (23.9)	**0.027**	1.22(1.02–1.46)
AA	896 (69.8)	967 (73.7)	N/A	1
*rs3818292*				
*Allele*				
G	724 (28.1)	740 (27.4)	0.583	0.97(0.86–1.09)
A	1852 (71.9)	1958 (72.6)	N/A	1
*Genotype*				
GG	102 (7.9)	100 (7.4)	0.585	1.09(0.81–1.46)
GA	520 (40.4)	540 (40.0)	0.761	1.03(0.87–1.20)
AA	666 (51.7)	709 (52.6)	N/A	1

Logistic regression analysis was used to compare alleles and genotypes between the T1D patients and healthy controls

*SNPs* Single-nucleotide polymorphisms, *CI* Confidence interval, *OR* Odds ratio, *N/A* Not applicable, *BMI* Body mass index

*P* < 0.05 was considered significant and is shown in bold

**Table 3:** Genetic models of rs10997866 and rs3818292 between cases and controls

SNP	Minor allele	Genetic model							
		Dominant model		Recessive model		Overdominant model		Additive model	
		OR (95% CI)	*P*	OR (95% CI)	*P*	OR (95% CI)	*P*	OR (95% CI)	*P*
rs10997866	G	1.21 (1.02–1.44)	**0.026**	0.92 (0.56–1.51)	0.733	1.23 (1.03–1.47)	**0.030**	1.22 (1.02–1.45)	**0.027**
rs3818292	G	1.04 (0.89–1.21)	0.663	1.09 (0.82–1.45)	0.574	1.02 (0.87–1.19)	0.857	0.98 (0.83–1.15)	0.761

Logistic regression analysis was used to compare genotypes between the T1D patients and healthy controls

*SNPs* single-nucleotide polymorphisms, *CI* confidence interval, *OR* odds ratio

### Associations of the two SNPs and the clinical characteristics of the T1D patients

We further evaluated whether a genotype–phenotype relationship for the *SIRT1* gene polymorphisms with the clinical characteristics of the T1D patients exists. The characteristics assessed included basic information (sex, age of onset, course of the disease, and BMI), biochemical measurements (FCP, PCP and HbA1c), and antibody results (GADA positivity rate and titer, IA-2A positivity rate and titer, and ZnT8A positivity rate). The results showed that both *SIRT1* rs10997866 and rs3818292 were significantly associated with the titer of IA-2A in T1D patients. Furthermore, the T1D patients with the GG genotype had higher IA-2A titers than those with the GA and AA genotypes for these two SNPs (*P* = 0.013 and *P* = 0.038, respectively, Tables 4 and 5).

**Table 4:** Clinical characteristics of T1D patients with different genotypes of rs10997866

Clinical characteristics	Genotype			*P* value
	GG	AG	AA	
Sample size	33	354	896	–
Sex (men/women)	19/14	186/168	488/408	0.759
Onset age (years)	21.0 (10.0, 33.7)	20.2 (11.0, 34.0)	22.0 (12.0, 34.0)	0.515
T1D duration (months)	2.0 (0.5, 11.7)	3.0 (0.5, 17.5)	3.0 (0.4, 14.0)	0.809
BMI (kg/m^2^)	18.75 ± 4.42	19.03 ± 3.49	19.07 ± 3.03	0.972
FBG (mmol/L)	8.50 (5.77, 12.11)	8.19 (6.20, 13.12)	8.75 (6.02, 13.40)	0.609
PBS (mmol/L)	13.70 (9.35, 19.97)	14.87 (10.20, 20.47)	15.60 (10.71, 20.93)	0.225
FCP (pmol/L)	100.75 (39.83, 235.78)	87.00 (33.47, 168.40)	87.00 (33.47, 168.40)	0.750
PCP (pmol/L)	160.00 (61.46, 345.04)	160.00 (61.46, 345.04)	160.00 (61.46, 345.04)	0.481
HbA1c (%)	8.95 (6.65, 12.18)	10.50 (7.90, 13.00)	10.15 (7.83, 12.80)	0.151
GADA positive (%)	96.97%	88.14%	90.71%	0.192
GADA titer (U/mL)	458.62 (95.89, 884.23)	315.90 (76.62, 784.03)	302.45 (78.38, 786.13)	0.365
IA-2A positive (%)	44.00%	54.55%	47.79%	0.135
IA-2A titer (U/mL)	476.79 (126.94, 1204.58)	202.49 (43.40, 727.45)	240.55 (54.16, 674.51)	0.013*
ZnT8A positive (%)	48.00%	36.63%	33.11%	0.224

The Kruskal‒Wallis H test and chi-square test were used to compare the characteristics of different genotypes among T1D patients. *P* < 0.05 was considered significant and is shown in bold

**Table 5:** Clinical characteristics of T1D patients with different genotypes of rs3818292

Clinical characteristics	Genotype			*P* value
	GG	GA	AA	
Sample size	102	520	666	–
Sex (men/women)	56/46	271/249	357/309	0.817
Onset age (years)	17.0 (10.5, 33.5)	23.0 (12.0, 35.0)	21.0 (11.0, 33.7)	0.251
T1D duration (months)	3.0 (0.4, 12.0)	3.0 (0.3, 13.0)	3.0 (0.5, 15.0)	0.778
BMI (kg/m^2^)	18.84 ± 4.74	19.22 ± 3.69	18.97 ± 3.56	0.530
FBG (mmol/L)	8.73 (5.97, 12.58)	8.39 (6.10, 12.99)	8.80 (6.18, 13.60)	0.719
PBS (mmol/L)	14.90 (10.35, 19.76)	15.20 (10.27, 20.42)	15.50 (10.59, 20.92)	0.773
FCP (pmol/L)	71.45 (21.63, 152.36)	91.25 (37.64, 168.78)	88.42 (30.47, 175.25)	0.264
PCP (pmol/L)	149.52 (50.13, 262.21)	169.53 (69.32, 345.37)	176.05 (67.66, 374.83)	0.186
HbA1c (%)	10.70 (7.80, 13.90)	10.20 (7.70, 12.90)	10.10 (7.90, 12.73)	0.519
GADA positive (%)	89.11%	89.79%	90.69%	0.823
GADA titer (U/mL)	250.98 (71.28, 768.14)	329.86 (102.71, 787.62)	332.96 (77.41, 823.33)	0.383
IA-2A positive (%)	51.16%	48.08%	50.47%	0.918
IA-2A titer (U/mL)	549.93 (88.62, 813.26)	231.99 (52.81, 673.15)	181.66 (33.62, 650.40)	0.038*
ZnT8A positive (%)	37.67%	37.80%	31.61%	0.163

The Kruskal‒Wallis H test and chi-square test were used to compare the characteristics of different genotypes among T1D patients. *P* < 0.05 was considered significant

## Discussion

There is growing evidence supporting the cytoprotective role of SIRT1 in diabetes and its complications [6, 19–21]. As an antioxidant, SIRT1 reduces oxidative stress (OS) in coronary arterial endothelial cells exposed to elevated glucose levels and then regulates insulin signaling [22]. A recent study demonstrated that varying expression levels of SIRT1 in diabetes may be partly attributed to OS [20]; this is consistent with early-stage diabetic rats showing decreased SIRT1 expression, with expression levels returning to normal after treatment with the antioxidant glucagon-like peptide 1 analog exendin-4 (EX4) [23]. In addition, downregulated SIRT1 has been reported to be associated with gestational diabetes and to play a possible role in reducing hypervascularization early in pregnancy and protecting against subsequent pregnancy complications caused by impaired placental growth [19].

Studies to date have mainly focused on the effects of altered SIRT1 protein expression on diabetes and related symptoms; nevertheless, as a complex autoimmune disease for which both genetic and environmental factors influence susceptibility [24], it has been widely accepted that genetic factors play an important role in the etiology of T1D [2, 24]. Indeed, a series of studies exploring the association between *SIRT1* gene polymorphisms in inflammation and autoimmune destruction have been carried out [17]. However, there is still minimal information about the role of *SIRT1* gene polymorphisms in the pathogenesis of T1D. Therefore, deeper exploration of the effect of *SIRT1* on T1D susceptibility from a genetic perspective is needed.

This case‒control study aimed to detect two selected *SIRT1* SNPs (rs10997866 and rs3818292) in the Han Chinese population with T1D to provide clues for elucidating the relationship between the *SIRT1* gene and T1D. Overall, our results showed that the *SIRT1* SNP rs10997866 is associated with an increased risk for T1D, with the G allele conferring higher risk. However, the association between T1D susceptibility and rs10997866 was observed for the GA genotype only, whereas no significant difference was detected for the GG genotype, possibly due to the relatively small sample size of GG genotype patients. Expanding the sample size may lead to statistically significant differences. Then, we found an association between rs10997866 and T1D susceptibility in genetic inheritance models (under dominant, overdominant and additive models). We also identified a link between these *SIRT1* SNPs and islet autoantibody titers in T1D patients, whereby T1D patients with the GG genotype of rs10997866 and rs3818292 had higher IA-2A titers than those with the GA and AA genotypes, suggesting an association between *SIRT1* risk alleles and IA-2A titers. These findings suggest that *SIRT1* risk variants might play a role in regulating autoimmunity and have the potential to be used as biomarkers for T1D progression.

To date, islet autoantibodies are still the most valuable biomarkers of islet autoimmunity, which indicates disease progression and diagnosis [25]. IA-2A has been identified as an independent predictor of more rapid progression to diabetes, especially when combined with GADA antibodies. Its longer prevalence time compared to other autoantibodies makes it a valuable tool in diagnosing latent autoimmune diabetes [26]. Individuals with IA-2A tend to be younger, have lower fasting and stimulated C-peptide levels, and experience a shorter duration of symptoms. Some immune-associated variants, such as in *IL27*, *IFIH1*, and *CTLA4*, have been linked to the presence of IA-2A [27–29]. However, there is significant variability in the rate of pancreatic β cell destruction, which may be caused by genetic susceptibility [26]. As it is believed that an individual's genotype remains constant from birth and is not influenced by age, any age discrepancies between cases and controls are not expected to impact results. Additionally, we conducted logistic regression analysis to assess whether differences in BMI influence the distribution of genotype and allele frequencies of the 2 SNPs. Our findings revealed no significant difference in genotype or allele frequency between the two groups (*P* > 0.05) after adjusting for BMI. Thus, we excluded the possibility that the observed differences were not due to age or BMI discrepancies.

Our study is the first to identify a correlation between *SIRT1* gene variants and increased T1D risk in a large sample of the Han Chinese population. However, there are some limitations. Importantly, in addition to genetic polymorphisms, the relationship between the *SIRT1* gene and T1D is complex and influenced by various factors; despite a reported positive association with this SNP and T2D or other autoimmune diseases, a correlation between *SIRT1* rs3818292 and T1D was not found in our study. We also analyzed the relationship between these *SIRT1* gene polymorphisms and clinical characteristics of T1D patients, but certain measurements assessing liver and renal function, such as blood lipids, liver and kidney function, and 24 h urinary albumin, were not obtained for all enrolled individuals. Furthermore, owing to the extensive temporal span encompassing our sample collection period, a subset of early-collected samples lacked the contemporaneous recording of insulin dosage administration. Consequently, we encountered obstacles in delineating insulin dosage utilization among individuals harboring distinct SIRT1 genotypes and its potential interplay with C-peptide levels within the purview of this investigation. Although our results suggested an association between *SIRT1* SNPs and T1D susceptibility, it is unclear whether these selected SNPs affect expression levels of SIRT1 and the functional role they play in the pathogenesis of T1D due to the lack of experimental data. Therefore, further research is required to investigate the biological mechanisms involved in the development of T1D.

## Conclusion

*SIRT1* rs10997866 is significantly associated with T1D susceptibility, and the minor allele G confers higher risk of T1D. Moreover, rs10997866 and rs3818292 in the *SIRT1* gene correlate with the rate of IA-2A positivity in Han Chinese individuals with T1D.

## Data Availability

The datasets analyzed during the current study are not publicly available but are available from the corresponding author on reasonable request.
